# Loss of DCC gene expression during ovarian tumorigenesis: relation to tumour differentiation and progression

**DOI:** 10.1054/bjoc.1999.0966

**Published:** 2000-01-18

**Authors:** M Saegusa, D Machida, I Okayasu

**Affiliations:** Department of Pathology, Kitasato University School of Medicine, 1-15-1 Kitasato, Sagamihara, Kanagawa, 228-8555, Japan

**Keywords:** DCC, p53, ovarian carcinoma, RT-PCR

## Abstract

To clarify the possible role of DCC gene alteration in ovarian neoplasias, we immunohistochemically investigated 124 carcinomas, as well as 55 cystadenomas and 41 low malignant potential (LMP) tumours and compared the results with those for p53 protein expression, clinicopathological factors and survival. A combination of the reverse transcription polymerase chain reaction (RT-PCR) and Southern blot hybridization (SBH) for DCC mRNA levels was also carried out on 26 malignant, five LMP, eight benign and seven normal ovarian samples. Significantly decreased levels of overall DCC values in carcinomas compared with benign and LMP lesions were revealed by both immunohistochemical and RT-PCR/SBH assays. Similar findings were also noted when subdivision was into serous and mucinous categories. In carcinomas, reduction or loss of DCC expression was significantly related to the serous phenotype (serous vs non-serous, *P*< 0.0001), a high histological grade (grade 1 vs 2 or 3, *P*< 0.02) and a more advanced stage (FIGO stage I vs II/III/IV, *P* = 0.0083), while no association was noted with survival. Although p53 immunopositivity demonstrated significant stepwise increase from benign through to malignant lesions, there was no clear association with DCC score values. The results indicated that impaired DCC expression may play an important role in ovarian tumorigenesis. In ovarian carcinomas, the altered expression is closely linked with tumour differentiation and progression. © 2000 Cancer Research Campaign


*British Journal of Cancer* (2000) **82**(3), 571–578
© 2000 Cancer Research Campaign
DOI: 10.1054/ bjoc.1999.0966, available online at http://www.idealibrary.com on IDEAL®

### Loss of DCC gene expression during ovarian tumorigenesis: relation to tumour differentiation and progression

**M Saegusa, D Machida and I Okayasu**

Department of Pathology, Kitasato University School of Medicine, 1-15-1 Kitasato, Sagamihara, Kanagawa 228-8555, Japan

**Summary** To clarify the possible role of DCC gene alteration in ovarian neoplasias, we immunohistochemically investigated 124 carcinomas, as well as 55 cystadenomas and 41 low malignant potential (LMP) tumours and compared the results with those for p53 protein expression, clinicopathological factors and survival. A combination of the reverse transcription polymerase chain reaction (RT-PCR) and Southern blot hybridization (SBH) for DCC mRNA levels was also carried out on 26 malignant, five LMP, eight benign and seven normal ovarian samples. Significantly decreased levels of overall DCC values in carcinomas compared with benign and LMP lesions were revealed by both immunohistochemical and RT-PCR/SBH assays. Similar findings were also noted when subdivision was into serous and mucinous categories. In carcinomas, reduction or loss of DCC expression was significantly related to the serous phenotype (serous vs non-serous, $P < 0.0001$), a high histological grade (grade 1 vs 2 or 3, $P < 0.02$) and a more advanced stage (FIGO stage I vs II/III/IV, $P = 0.0083$), while no association was noted with survival. Although p53 immunopositivity demonstrated significant stepwise increase from benign through to malignant lesions, there was no clear association with DCC score values. The results indicated that impaired DCC expression may play an important role in ovarian tumorigenesis. In ovarian carcinomas, the altered expression is closely linked with tumour differentiation and progression. © 2000 Cancer Research Campaign

**Keywords:** DCC; p53; ovarian carcinoma; RT-PCR

The DCC (deleted in colon carcinoma) gene, a putative tumour suppressor gene, spans nearly 1.35 megabases with at least 29 exons and is located within the 18q21.3 region (Fearon et al, 1990). The cDNA predicts a 1447-amino acid transmembrane protein with four immunoglobulin-like and six fibronectin III-like domains, showing high homology to the neural cell adhesion molecule family of cell surface proteins (Cho et al, 1994). In normal mammalian tissues, abundant expression of DCC mRNA and protein is common in the central and peripheral nervous system, as well as most epithelial tissues (Hedrick et al, 1994; Reale et al, 1994). Full-length but not truncated DCC was found to inhibit tumorigenicity in a human squamous cell carcinoma cell line lacking endogenous DCC expression (Klingelhutz et al, 1995), and reduced or missing DCC expression has been demonstrated in a variety of human malignancies, including prostate, gastric and oesophageal carcinomas (Gao et al, 1993; Huang et al, 1992; Uchino et al, 1992).

The ovarian epithelial carcinoma is clinically one of the most important tumours of the female reproductive system, since approximately 75% of patients are diagnosed at an advanced stage. Recent molecular studies of ovarian neoplasias have demonstrated the presence of several genetic alterations, such as CD44 abnormalities, c-*erb*B-2 amplification, and K-*ras* mutations (Slamon et al, 1989; Zeimet et al, 1997; Mandai et al, 1998). Although DCC expression has been investigated using a limited number of ovarian carcinoma cases (Enomoto et al, 1995), any association with tumour progression or survival is still unclear. In addition, little is known about changes in benign and premalignant ovarian tumours.

The p53 gene is also a tumour suppressor gene, and a high incidence of gene mutations leading to non-functional protein overexpression has been demonstrated in various types of human malignant tumours, including ovarian carcinomas (Herod et al, 1996). However, there have been no reports concerning any relation between p53 and DCC alterations in ovarian tumours.

In this study, we investigated benign, premalignant and malignant ovarian neoplasias to clarify the possible role of DCC gene expression in tumour development and progression, using immunohistochemistry and a combination of the reverse transcription polymerase chain reaction (RT-PCR) assay with Southern blot hybridization (SBH). The results were compared with p53 expression, several prognostic factors and survival.

### MATERIALS AND METHODS

### Cases

A total of 124 cases of ovarian carcinomas, surgically resected at Kitasato University Hospital during the period from 1992 to 1998, were investigated, along with 41 low malignant potential (LMP) tumours and 55 cystadenomas. The ages of the patients ranged from 26 to 83 years, with a mean of 53.6.

All cases underwent oophorectomy with or without hysterectomy. All tissues were routinely fixed in 10% formalin and processed for embedding in paraffin wax. Histological diagnoses were performed according to the criteria of the International

*Received 8 December 1998*
*Revised 28 June 1999*
*Accepted 3 August 1999*

*Correspondence to:* M Saegusa

Federation of Gynecology and Obstetrics (FIGO) and the WHO international system. The tumour cases investigated comprised 51 serous, 23 mucinous, 13 endometrioid and 37 clear cell ovarian carcinomas, as well as 12 serous and 29 mucinous LMP tumours, and 22 serous and 33 mucinous cystadenomas. Data for clinicopathological factors, including histological grade, FIGO stage and lymph node status, were also examined for comparison with the results of immunohistochemistry.

Of 124 ovarian carcinoma cases, 106 could be analysed for their outcome after surgery, with a mean follow-up time of 22.7 months (range 1–75 months). Some cases had received platinum-based chemotherapy after primary surgery.

Twenty-six ovarian carcinoma (12 serous, two endometrioid, five mucinous and seven clear cell), five LMP (one serous and four mucinous), eight cystadenoma (two serous and six mucinous), and seven normal ovarian tissue samples were snap-frozen in liquid nitrogen for DCC mRNA analysis.

### Immunohistochemistry

Immunohistochemistry was performed using a combination of microwave-oven heating and standard streptavidin–biotin–peroxidase complex (LSAB kit, Dako, Copenhagen, Denmark) methods. Briefly, slides were heated in 10 mM citrate buffer (pH 6.0) for six 5-min cycles for DCC proteins, as described by Shibata et al (1996), and three 5-min cycles for p53 proteins, using a microwave oven and then incubated overnight at 4°C with optimum dilutions of primary antibodies. The antibodies used were anti-human DCC monoclonal antibody (clone G97-449, ×100 dilution, Pharmingen, San Diego, CA, USA) and anti-p53 protein (DO-7) mouse monoclonal antibody (×500 dilution, Novocastra Lab. Ltd, Newcastle, UK). To confirm the specificity of binding, normal mouse serum (×500 dilution) was supplied instead of primary antibody as a negative control.

### Evaluation for DCC and p53 immunoreactivity

Scoring of the DCC immunohistochemistry results was achieved as previously reported (Sinicrope et al, 1995; Saegusa et al, 1996). Briefly, based on the percentages of immunopositive epithelial cells among the totals of normal or neoplastic cells on a slide section, subdivision was made into five categories as follows: 0, all negative; 1, < 10% positive cells; 2, 10–30%; 3, 30–50%; and 4, > 50%. The immunointensity was also subclassified into four groups as follows: 0, negative; 1+, weak; 2+, moderate; and 3+, strong. Immunoreactivity scores were generated by multiplication of the values for the two parameters. As positive controls, normal colonic epithelium for DCC and a p53-positive colorectal carcinoma for p53 were used.

With regard to p53, the staining results were considered to be positive when more than 30% of the totals of neoplastic cells showed nuclear staining (Baas et al, 1994).

### Reverse transcription-polymerase chain reaction

Total cellular RNA was extracted from 40 neoplastic and seven normal ovarian frozen tissues using Isogen (Nippon Gene Co., Tokyo, Japan) and cDNAs were synthesized from 5 µg of total RNA using RAV-2 reverse transcriptase (Takara, Shiga, Japan) in the presence of random primers (Takara) and a ribonuclease inhibitor (Takara) in a 20 µl reaction volume at 42°C for 60 min. One microlitre of cDNA solution was amplified by *Taq* polymerase (Takara) in a volume of 10 µl. For detection of DCC mRNA expression in exons 6–7, primers used were 5′- TTCCGCCATGGTTTTTAAATCA-3′ (sense) and 5′-AGCCTCATTTTCAGCCACACA-3′ (anti-sense), as reported by Fearon et al (1990). For analysis of alternative splicing in the extracellular domain (exon 17), the primers used were DCK2834S, 5′-CCCAGACTAACTGCATCATCATGAG-3′ (sense), and DCK3151A, 5′-CACCTACTGGTGGGAGCAT-3′ (anti-sense), described by Reale et al (1994). The PCR procedure was performed with 35 cycles of denaturation at 94°C for 0.5 min, annealing at 55°C for 1 min and extension at 72°C for 1 min, with a final extension time of 7 min. As a negative control, water was supplied instead of template DNA for each examination. To examine the quality and quantity of the synthesized cDNA, the β-actin specific primers (sense, 5′–TGCTATCCAGGCTGTGCTAT-3′ and anti-sense, 5′-GATGGAGTTGAAGGTAGTTT-3′) were also applied for amplification (Horstmann et al, 1997). PCR assays were performed in duplicate or triplicate.

### Southern blot hybridization

A 10 µl aliquot of each PCR reaction mixture was electrophoresed in a 3.0% agarose gel and transferred to a Hybond N nylon membrane (Amersham, Tokyo, Japan). After prehybridization using DIG Easy Hyb (Boehringer Mannheim, Tokyo, Japan) solution, filters were hybridized overnight with each digoxigenin-labelled exon-specific probe, which corresponded to internal sites between primer sets used. The sequences of oligonucleotide probes for DCC exon 6/7, DCC exon 17, and β-actin were as follows: probe DCC exon 6/7 (5′-AATTGGATGAAGAATGGAGATGTGGTCATT-3′, encoding nucleotides 1087–1116 in the cDNA sequence), probe DCC exon 17 (5′-ATGAGTTGGACTCCTCCCTTGAAC-3′, nucleotides 2226–2250), and probe β-actin (5′-ACTGACTACCTCATGAAGATCCTCACCGAG-3′, nucleotides 597–626). Hybridization signals were detected with a DIG Luminescent Detection Kit (Boehringer Mannheim). The conditions used for hybridization, washing and detection were in line with the manufacturer’s recommendations. Between each hybridization the filter was stripped before being rehybridized with another probe.

### RT-PCR/SBH data analysis

Quantitation of hybridization signals for DCC exons 6/7 and 17, and β-actin, respectively, was performed by densitometric analysis using NIH Image version 1.58 software. The relative expression level of DCC mRNA was calculated by normalization to the hybridization signals for β-actin in each case: the value for DCC mRNA signal was divided by that for β-actin. Based on the average values for relative DCC transcripts in normal samples, subdivision was into four categories as follows: negative, <10% of average values for normal ovaries; ±, 10–50%; 1+, 50–100%; 2+, >100%, according to the methods described by Enomoto et al (1995), with minor modification. A loss or reduction of DCC expression was concluded with scores of either negative or ±, and a positive one with 1+ or 2+.

Detection of alternative splicing within DCC exon 17 was performed as described by Reale et al (1994). Briefly, DCK2834S

© 2000 Cancer Research Campaign

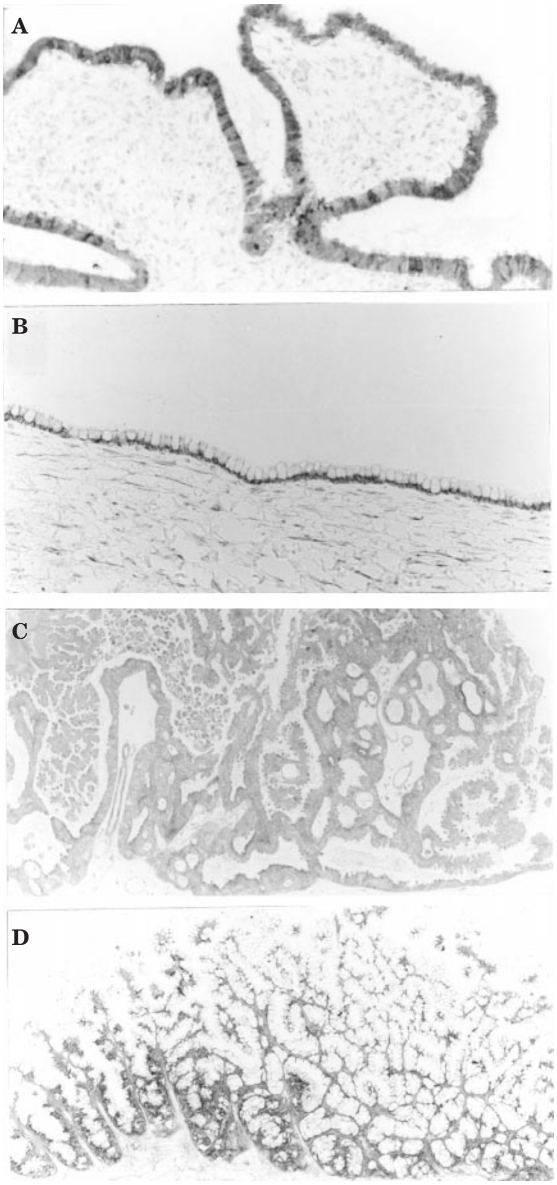

**Figure 1** DCC immunoreactivity. (**A, B**) Cystadenomas of serous (**A**) and mucinous (**B**) types. Original magnification, ×200. (**C, D**) Low malignant potential tumours of serous (**C**) and mucinous (**D**) types. Original magnification, ×100. Note the moderate to strong immunoreactivity in both lesions

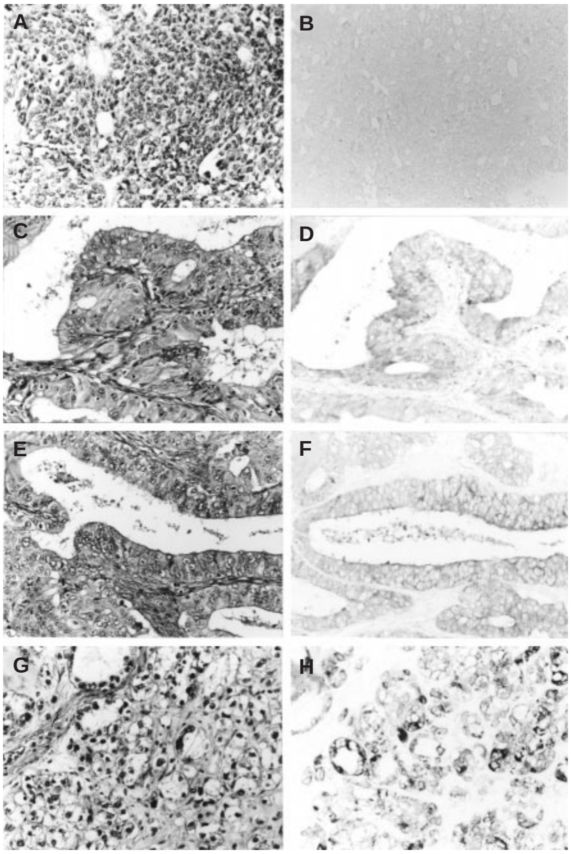

**Figure 2** Semiserial sections of ovarian carcinomas of grade (**G**) 3 serous (**A, B**), G1 mucinous (**C, D**), G1 endometrioid (**E, F**), and G2 clear cell (**G, H**) types. (**A, C, E, G**) H&E staining. (**B, D, F, H**) Immunohistochemistry for DCC protein. A negative reaction is evident in the serous carcinoma (**B**), while the other tumours are variously positive. Original magnification, ×200

and DCK3151A primers generated a product of 341 bp if the more 5′ splice acceptor at exon 17 was used (60-nucleotide sequence present) or an alternatively spliced product of 281 bp if the more 3′ splice acceptor site was employed.

### Statistics

Data for DCC and p53 immunoreactivity were statistically analysed using the Mann–Whitney *U*-test, the Pearson's correlation coefficient, and the $\chi^2$ test.

Survival was measured from the time of the primary operation and survival curves were generated by the methods of Kaplan and Meier. The log-rank test and Cox proportional hazards modelling were performed to compare survival rates between subgroups classified on the basis of clinicopathological data and immunoreactivity for DCC and p53. The cut-off for statistical significance was defined as $P < 0.05$.

### RESULTS

### DCC protein expression

In normal ovarian tissues, weak to moderate DCC immunoreactivity was observed in surface epithelial cells and stromal cells. In mature follicles, theca cells were positive but not granulosa cells. In addition, moderate to strong immunoreactivity was apparent in Walthard's nests.

Moderate to strong cytoplasmic staining for DCC was diffusely observed in cystadenomas and LMP tumours, whereas variability in immunointensity and positive cell distribution was revealed for carcinomas (Figures 1 and 2).

Overall average DCC values demonstrated significant stepwise decrease from cystadenomas through to carcinomas, in line with the results for serous and mucinous categories (Figure 3). In ovarian carcinomas, significant differences for DCC scores among histological phenotypes (serous vs non-serous, $P < 0.0001$), histological grades (G1 vs G2 or G3), and clinical stages (FIGO stage I vs II/III/IV, $P = 0.0083$) were observed. Regarding lymph node metastasis, although higher DCC scores were found for negative

*© 2000 Cancer Research Campaign*

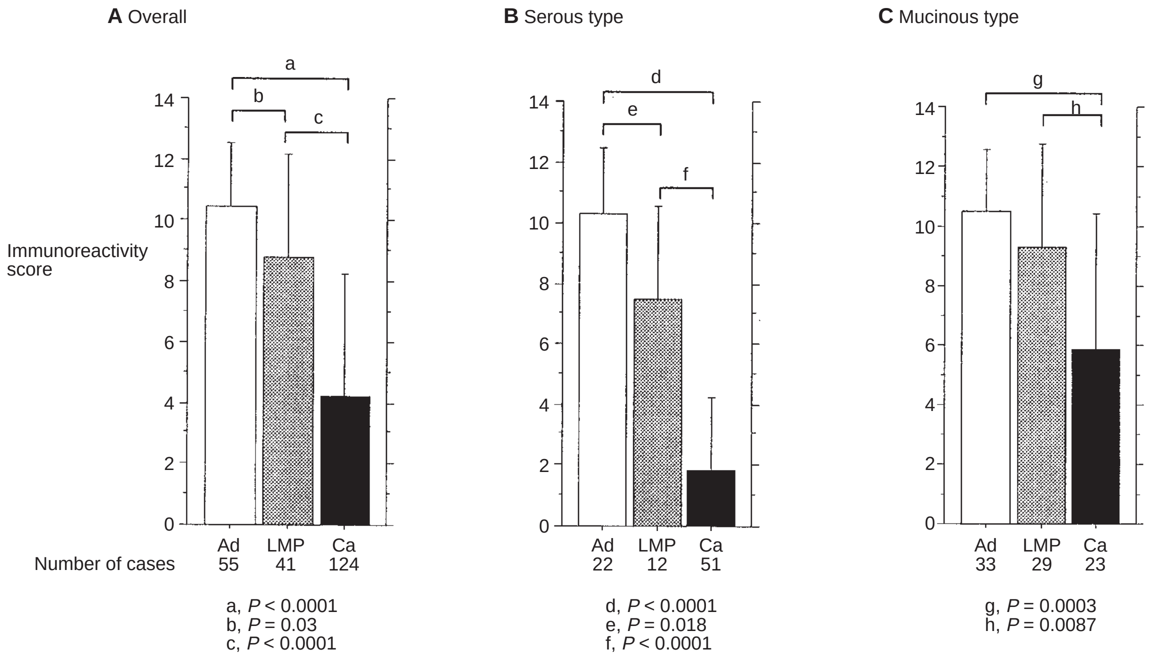

**Figure 3** (**A**) Overall immunoreactivity score values for DCC in cystadenoma (Ad), low malignant potential (LMP), and carcinoma (Ca) lesions. (**B**) DCC scores in serous type ovarian tumours. (**C**) DCC scores in mucinous type ovarian tumours. The data are mean ± s.d. values

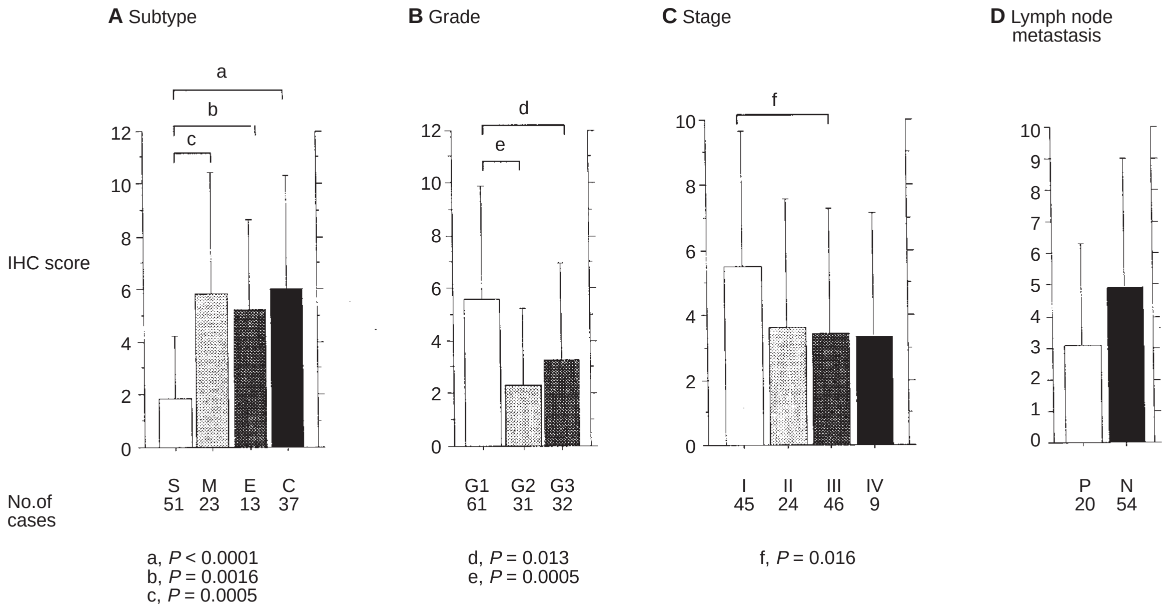

**Figure 4** Relation between ovarian carcinoma type and DCC immunoreactivity scores and clinicopathological factors. S, serous type; M, mucinous type; E, endometrioid type; C, clear cell type; G, grade; P, positive; N, negative. The data are mean ± s.d. values as compared to positive cases, the difference did not reach significance ($P = 0.093$) (Figure 4).

### DCC mRNA analysis

RNAs obtained from all ovarian samples could be successfully amplified using primer sets for the β-actin gene, providing a fragment with a molecular weight of 446 bp.

With primer sets corresponding to DCC exons 6–7 and 17, RT-PCR products were observed with molecular weights of 233 bp and 341 bp, respectively, a positive correlation ($r = 0.874$, $P < 0.0001$) between the relative amounts of both amplicons for all categories being significant. An alternative spliced product of 281 bp in exon 17 was detected in only one ovarian carcinoma (case 5), while none of non-malignant samples demonstrated such signals (Figure 5).

*© 2000 Cancer Research Campaign*

**Table 1** Data for DCC mRNA analysis in ovarian neoplasias

| No. | Hist | Grade | Stage | LN status | IHC for p53 | mRNA (DCC/β actin)* Exon 6/7 | Exon 17 | DCC score |
|---|---|---|---|---|---|---|---|---|
| Carcinoma | | | | | | | | |
| C1 | S | G3 | II | P | N | 1.38 (2+) | 1.14 (2+) | 3 |
| C2 | S | G1 | III | N | P | 1.72 (2+) | 1.56 (2+) | 4 |
| C3 | E | G1 | I | NE | N | 1.64 (2+) | 1.08 (2+) | 6 |
| C4 | C | G2 | II | P | P | 0.89 (1+) | 0.5 (1+) | 0 |
| C5 | C | G3 | I | N | N | 0.23 (±) | 0#(–) | 8 |
| C6 | C | G2 | I | N | N | 0.78 (1+) | 0.7 (1+) | 0 |
| C7 | E | G1 | II | N | N | 0.83 (1+) | 0.73 (1+) | 4 |
| C8 | M | G1 | I | N | P | 0 (–) | 0.19 (±) | 0 |
| C10 | S | G1 | I | N | ND | 0.85 (1+) | 1 (2+) | 6 |
| C11 | S | G1 | III | P | P | 0 (–) | 0.22 (±) | 3 |
| C12 | S | G3 | III | P | P | 1.23 (2+) | 0.87 (1+) | 3 |
| C13 | S | G3 | III | NE | P | 1.06 (2+) | 0.84 (1+) | 0 |
| C14 | C | G1 | II | N | N | 0.81 (1+) | 0.75 (1+) | 6 |
| C15 | M | G3 | III | P | N | 0 (–) | 0 (–) | 0 |
| C16 | S | G1 | III | NE | P | 0.24 (±) | 0.52 (1+) | 0 |
| C17 | S | G1 | II | NE | P | 0.16 (±) | 0.58 (1+) | 0 |
| C18 | S | G2 | II | N | P | 1.22 (2+) | 1.52 (2+) | 4 |
| C19 | S | G3 | IV | NE | N | 1.48 (2+) | 2 (2+) | 2 |
| C20 | C | G3 | I | N | P | 1 (2+) | 0.93 (2+) | 12 |
| C21 | M | G2 | III | NE | ND | 0.14 (±) | 0 (–) | 0 |
| C22 | S | G2 | III | NE | N | 0.38 (±) | 0.83 (1+) | 2 |
| C23 | S | G2 | II | NE | P | 1.33 (2+) | 1.46 (2+) | 0 |
| C24 | C | G1 | I | N | N | 0 (–) | 0 (–) | 0 |
| C25 | M | G1 | I | N | P | 0.5 (1+) | 0.71 (1+) | 12 |
| C26 | M | G1 | I | N | ND | 1.33 (2+) | 1.58 (2+) | 8 |
| C27 | C | G1 | I | N | P | 0 (–) | 0 (–) | 0 |
| LMP tumours | | | | | | | | |
| L1 | M | | | | P | 0.7 (1+) | 0.78 (1+) | 8 |
| L2 | S | | | | N | 0.93 (2+) | 0.75 (1+) | 8 |
| L3 | M | | | | N | 1.07 (2+) | 1 (2+) | 12 |
| L4 | M | | | | N | 0.52 (1+) | 0.67 (1+) | 8 |
| L5 | M | | | | N | 1.32 (2+) | 1.32 (2+) | 12 |
| Cystadenomas | | | | | | | | |
| A1 | S | | | | N | 0.74 (1+) | 0.89 (1+) | 8 |
| A2 | M | | | | N | 0.91 (2+) | 0.81 (1+) | 12 |
| A3 | M | | | | N | 0.79 (1+) | 0.85 (1+) | 9 |
| A4 | M | | | | N | 0.94 (2+) | 0.9 (1+) | 12 |
| A5 | S | | | | N | 1 (2+) | 0.97 (2+) | 8 |
| A6 | M | | | | N | 1.09 (2+) | 1.27 (2+) | 9 |
| A7 | M | | | | N | 0.75 (1+) | 0.42 (±) | 12 |
| A8 | M | | | | N | 1.75 (2+) | 1.15 (2+) | 12 |
| Normal (*n* = 7) | | | | | | 0.9±0.3 | 0.9±0.4 | |

Hist, histology; S, serous type; E, endometriod type; M, mucinous type; c, Clear cell type; LMP, low malignant potential; LN meta, lymph node metastasis; IHC, immunohistochemistry; P, positive; N, negative; ND, not done; *, relative intensity and score of DCC mRNA; #, alternatively spliced product.

Data from DCC mRNA analysis of ovarian carcinomas are summarized in Table 1. Reduction (±) or loss (–) of the expression for either DCC exons 6/7 or 17 transcripts was observed in ten (34.5%) of 26 carcinomas. The average DCC immunoreactivity scores (1.3 ± 2.6, mean ± s.d.) for lesions with impaired mRNA expression were significantly lower than those (4.6 ± 3.8) in the non-altered group ($P < 0.02$). There was no association between altered DCC mRNA levels and any of several clinicopathological factors investigated.

### p53 protein expression

Nuclear p53 staining was sporadically observed in cystadenomas, while diffusely distributed strong immunoreactivity was revealed in carcinomas. In LMP tumours, the immunointensity and the distribution indicated an intermediate status. None of the normal ovarian elements showed any immunoreaction.

The p53 positivity was significantly increased in the sequence leading from benign to malignant lesions. In carcinomas, the immunoreactivity was significantly related to histological subtypes (serous vs non-serous), but there was no association with histological grade, clinical stage, lymph node status, or DCC score (Table 2).

### Survival

To examine the relation between DCC protein expression and survival in ovarian carcinomas, subdivision was made into two categories of non-altered (score ≥ 3) and impaired (score ≤ 2) groups, on the basis of average values of immunohistochemical

© 2000 Cancer Research Campaign 

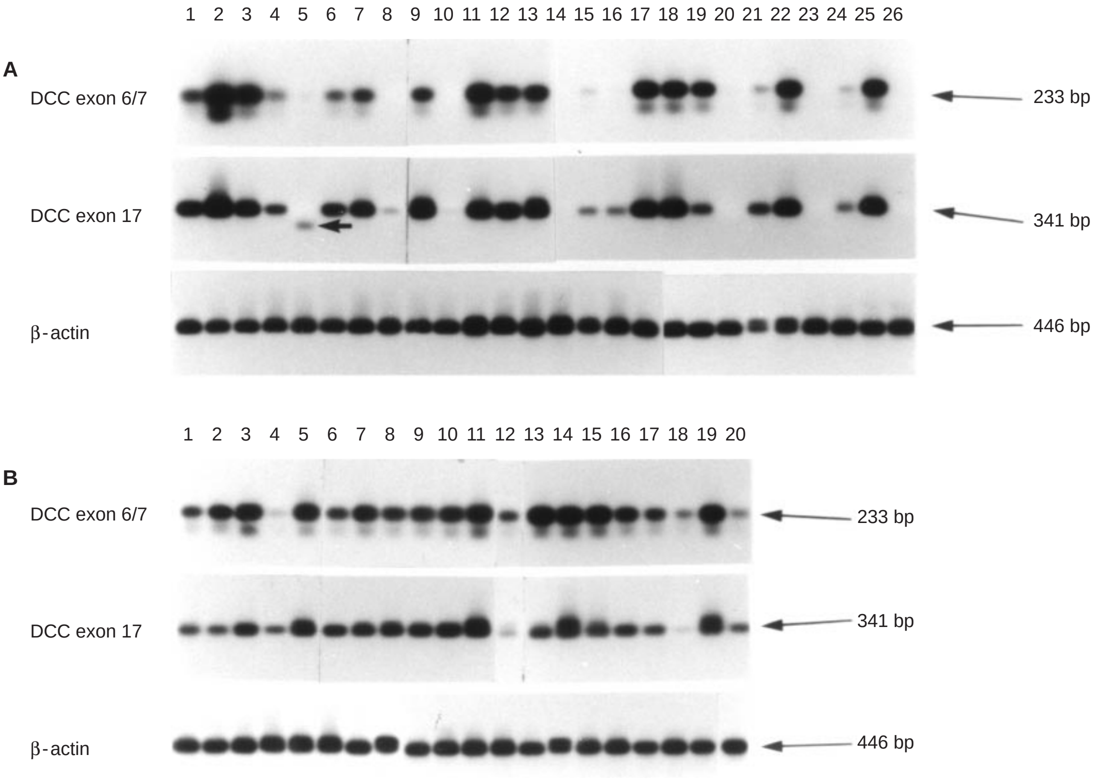

**Figure 5** RT-PCR/Southern blot hybridization assay for DCC and β-actin in ovarian carcinomas (**A**) and non-malignant ovarian samples, including normal, benign, and premalignant lesions (**B**). (**A**) Case 5 (lane 5) shows an alternative spliced product in DCC exon 17 (indicated by arrow). (**B**) Lanes 1–5, low malignant potential tumours; lanes 6–13, cystadenomas; lanes 14 to 20, normal ovarian samples. Case numbers correspond to those in Table 1

**Table 2** p53 expression in benign, premalignant and malignant ovarian neoplasias

| | *n* | p53 immunopositivity | *P*-value |
|---|---|---|---|
| Cystadenoma | 40 | 1 (2.5%) | |
| LMP tumour | 36 | 11 (30.6%) | |
| Ovarian carcinoma | 124 | 57 (46.0%) | <0.0001 |
| Subtype | | | |
| Serous | 51 | 36 (70.6%) | |
| Non-serous | 73 | 17 (23.3%) | <0.0001 |

LMP, low malignant potential.

DCC scores (4.2 ± 4.0) and the results of mRNA analysis of ovarian carcinomas. As shown in Table 3, the results of univariate analysis for prognostic markers by log-rank test in overall survival times revealed prognostic significance for the FIGO stage, lymph node status and DCC score in p53 negative group. On multivariate analysis, however, these parameters did not retain statistical significance (data not shown).

**Table 3** Univariate analysis of prognostic factors for ovarian carcinomas

| Factor | Category | *n* | Log-rank c2 | *P*-value |
|---|---|---|---|---|
| Subtype | Serous | 41 | 1.41 | 0.23 |
| | Non-serous | 52 | | |
| Grade | G1 | 55 | 0.83 | 0.36 |
| | G2/G3 | 51 | | |
| FIGO stage | Stage I | 38 | 5.49 | 0.02 |
| | Stage II/III/IV | 68 | | |
| Lymph node metastasis | Positive | 18 | 9.33 | 0.002 |
| | Negative | 46 | | |
| p53 status | Positive | 47 | 0.17 | 0.68 |
| | Negative | 58 | | |
| DCC score | ≥3 | 63 | 1.4 | 0.29 |
| | 2≥ | 43 | | |
| p53 (+) category | DCC score ≥3 | 26 | 0.34 | 0.56 |
| | DCC score 2≥ | 21 | | |
| p53 (–) group | DCC score ≥3 | 36 | 4.13 | 0.04 |
| | DCC score 2≥ | 22 | | |

### DISCUSSION

Little is known about the role of DCC gene in regulation of normal cell growth or differentiation. Given several similarities between DCC and members of the N-CAM family, it is speculated that DCC may contribute to cell–cell or cell–extracellular matrix interaction during cellular differentiation and development (Hedrick et al, 1994). Chuong et al (1994) have considered that DCC is required to maintain the basal cell status, proliferating but under control, ready to respond to differentiating signals. Keino-Masu et al (1996) have proposed that DCC is a receptor or a component of a receptor that mediates the effects of netrin-1 on commissural axons. In this study, certain levels of DCC mRNAs and proteins were detected in normal ovarian tissues, suggesting that DCC may also play some role in controlling differentiation of some components in normal ovary.

 *© 2000 Cancer Research Campaign*

It has been considered that ovarian epithelial carcinomas develop from the surface epithelium or inclusion cysts directly (de novo) or from pre-existing benign epithelial lesions (Bell and Scully, 1994). LMP tumours are clearly distinguished from ovarian carcinomas by their indolent clinical outcome and delayed recurrence (DiSaia and Creasman, 1993). Under pathological conditions, if DCC is still present, increased cell proliferation may be kept under control, whereas loss of DCC in these cells might lead to loss of cell interaction, loss of proliferation control and cell dispersion (Chuong et al, 1994). In our study, significant decrease of DCC expression was noted from benign through to malignant lesions, even when subdivision was into serous and mucinous phenotypes.

In colorectal carcinomas, DCC expression has been shown to be reduced during tumour progression from intramucosal to invasive carcinomas, suggesting a functional role as a suppressor of metastasis (Kikuchi-Yanoshita et al, 1992). Reyes-Mugica et al (1997) have demonstrated DCC inactivation in glioma progression, suggesting that the expression is preferentially, but not exclusively, lost in the genetic pathway to secondary glioblastoma multiforme. In our large series of ovarian carcinomas, the reduction or loss of the DCC immunoreactivity was closely related to a higher histological grade and a more advanced clinical stage, indicative of a linkage with malignant potential. Enomoto et al (1995) also described similar findings using 22 ovarian carcinomas. With regard to a lack of such association on mRNA analysis, one reason may be due to the relatively small numbers of cases investigated.

It is widely accepted that overexpression of p53 proteins detected by immunohistochemistry is usually due to an underlying mutation of the p53 gene, although p53 expression may not always indicate the presence of gene mutations (Maestro et al, 1992; Wynford-Thomas, 1992). Baas et al (1994) have earlier described that high expression (labelling index above 30%) is highly specific (90%) to mutated cases. In this study, p53 protein accumulation was detected in 46.0% of ovarian carcinomas with a particular predominance in those of serous phenotype, in line with previous studies demonstrating p53 gene mutations or overexpression in approximately 50% of ovarian carcinomas (Klemi et al, 1995; Reles et al, 1996; Eltabbakh et al, 1997). In contrast, our finding of p53 overexpression in 30.6% of LMP tumours appears to be higher as compared to previous investigations (Inoue et al, 1994; Klemi et al, 1994). Although the exact reason for the discrepancy with the literature is unclear, one possibility may be due to differences in the antibodies used or criteria for conclusion of positive immunoreactivity.

Previous studies of the relation between p53 alterations and survival of ovarian cancer cases have produced conflicting results. Some investigators demonstrated that p53 abnormalities are significantly associated with a poor prognosis (Klemi et al, 1995; Herod et al, 1996), whereas others could not confirm such results (Reles et al, 1996; Eltabbakh et al, 1997). In our cases, p53 overexpression did not correlate with survival or any of the clinicopathological parameters. Although it has been reported that gastric carcinomas with loss of heterozygosity at DCC locus frequently exhibited p53 gene mutations, even in their early stage (Maezawa et al, 1995), our results did not show such association.

Shibata et al (1996) have provided immunohistochemical evidence of a prognostic significance of DCC expression in stage II or III colorectal carcinomas. Goi et al (1998) also indicated that colonic cancer patients with liver metastasis expressed significantly lower levels of DCC protein than those without. In this study, however, we could not demonstrate the prognostic values of DCC expression in ovarian carcinomas. Although low DCC scores were associated with unfavourable survival in the p53 negative carcinoma group by univariate analysis, the significance of this is unclear.

Molecular mechanisms of tumour suppressor gene inactivation have been described at the DNA level, including point mutations, chromosomal deletions, rearrangements and insertions. Specific mutations in the DCC gene have not been identified, implying that other mechanisms, such as allelic loss, aberrant splicing of transcripts, and allele-specific loss of transcripts, contribute to the inactivation of the gene (Ekstrand et al, 1995; Kong et al, 1997). Alternative splicing sites of DCC transcripts have been demonstrated in extracellular (exon 17) and cytoplasmic (exon 26) domain sequences (Reale et al, 1994). In this study, an alternative splicing transcript resulting in an extracellular domain change was only observed in one (3.8%) carcinoma case. Although we did not examine the cytoplasmic domain sites, our results suggested that in ovarian carcinomas alternative splicing may play a minor role in inactivation of DCC gene expression.

In conclusion, we have demonstrated that impairment of DCC expression specifically occurs in ovarian carcinomas. In ovarian carcinomas, the altered expression is closely linked with tumour differentiation and progression.

### REFERENCES

Baas IO, Mulder J-WR, Offerhaus JA, Vogelstein B and Hamilton SR (1994) An evaluation of six antibodies for immunohistochemistry of mutant p53 gene product in archival colorectal neoplasms. *J Pathol* **172**: 5–12

Bell DA and Scully RE (1994) Early de novo ovarian carcinoma: a study of fourteen cases. *Cancer* **73**: 1859–1864

Cho KR, Oliner JD, Simons JW, Hedrick L, Fearon ER, Preisinger AC, Hedge P, Silverman GA and Vogelstein B (1994) The DCC gene: structural analysis and mutations in colorectal carcinomas. *Genomics* **19**: 525–531

Chuong C-M, Jiang T-X, Yin E and Widelitz RB (1994) cDCC (chicken homologue to a gene deleted in colorectal carcinoma) is an epithelial adhesion molecule expressed in the basal cells and involved in epithelial–mesenchymal interaction. *Dev Biol* **164**: 383–397

DiSaia PJ and Creasman WT (1993) Borderline malignant epithelial ovarian cancer. In: *Clinical Gynecologic Oncology*, Manning S (ed), pp. 320–323. Mosby-Year Book: St Louis

Ekstrand BC, Mansfield TA, Bigner SH and Fearon ER (1995) DCC expression is altered by multiple mechanisms in brain tumours. *Oncogene* **11**: 2393–2402

Eltabbakh GH, Belinson JL, Kennedy AW, Biscotti CV, Casey G, Tubbs RR and Blumenson LE (1997) p53 overexpression is not an independent prognostic factor for patients with primary ovarian epithelial cancer. *Cancer* **80**: 892–898

Enomoto T, Fujita M, Cheng C, Nakashima R, Ozaki M, Inoue M and Nomura T (1995) Loss of expression and loss of heterozygosity in the DCC gene in neoplasms of the human female reproductive tract. *Br J Cancer* **71**: 462–467

Fearon ER, Cho KR, Nigro JM, Kern SE, Simons JW, Ruppert JM, Hamilton SR, Preisinger AC, Thomas G, Kinzler KW and Vogelstein B (1990) Identification of a chromosome 18q gene that is altered in colorectal cancer. *Science* **247**: 49–56

Gao X, Honn KV, Grignon D, Sakr W and Chen YQ (1993) Frequent loss of expression and loss of heterozygosity of the putative tumor suppressor gene DCC in prostatic carcinomas. *Cancer Res* **53**: 2723–2727

Goi T, Yamaguchi A, Nakagawara G, Urano T, Shiku H and Furukawa K (1998) Reduced expression of deleted colorectal carcinoma (DCC) protein in established colon cancers. *Br J Cancer* **77**: 466–471

Hedrick L, Cho KR, Fearon ER, Wu T-C, Kinzler KW and Vogelstein B (1994) The DCC gene product in cellular differentiation and colorectal tumorigenesis. *Genes Dev* **8**: 1174–1183

Herod JJO, Eliopoulos AG, Warwick J, Niedobitek G, Young LS and Kerr DJ (1996) The prognostic significance of bcl-2 and p53 expression in ovarian carcinoma. *Cancer Res* **56**: 2178–2184

© 2000 Cancer Research Campaign

Horstmann MA, Posl M, Scholz RB, Anderegg B, Simon P, Baumgaertl K, Delling G and Kabisch H (1997) Frequent reduction or loss of DCC gene expression in human osteosarcoma. *Br J Cancer* **75**: 1309–1317

Huang Y, Boynton RF, Blount PL, Silverstein RJ, Yin J, Tong Y, McDaniel TK, Newkirk C, Resau JH, Sridhara R, Reid BJ and Meltzer SJ (1992) Loss of heterozygosity involves multiple tumor suppressor genes in human esophageal cancers. *Cancer Res* **52**: 6525–6530

Inoue M, Fujita M, Enomoto T, Morimoto H, Monden T, Shimano T and Tanizawa O (1994) Immunohistochemical analysis of p53 in gynecologic tumors. *Am J Clin Pathol* **102**: 665–670

Keino-Masu K, Masu M, Hinck L, Leonardo ED, Chan SS-Y, Culotti JG and Tessier-Lavigne M (1996) Deleted in colorectal cancer (DCC) encodes a netrin receptor. *Cell* **87**: 175–185

Kikuchi-Yanoshita R, Konishi M, Fukunari H, Tanaka K and Miyaki M (1992) Loss of expression of the DCC gene during progression of colorectal carcinomas in familial adenomatous polyposis and non-familial adenomatous polyposis patients. *Cancer Res* **52**: 3801–3803

Klemi P-J, Takahashi S, Joensuu H, Kiilholma P, Narimatsu E and Mori M (1994) Immunohistochemical detection of p53 protein in borderline and malignant serous ovarian tumors. *Int J Gyne Pathol* **13**: 228–233

Klemi P-J, Pylkkanen L, Kiilholma P, Kurvinen K and Joensuu H (1995) p53 protein detected by immunohistochemistry as a prognostic factor in patients with epithelial ovarian carcinoma. *Cancer* **76**: 1201–1208

Klingelhutz AJ, Hedrick L, Cho KR and McDougall JK (1995) The DCC gene suppresses the malignant phenotype of transformed human epithelial cells. *Oncogene* **10**: 1581–1586

Kong X-T, Choi SH, Inoue A, Xu F, Chen T, Takita J, Yokota J, Bessho F, Yanagisawa M, Hanada R, Yamamoto K and Hayashi Y (1997) Expression and mutational analysis of the DCC, DPC4, and MADR2/JV18-1 genes in neuroblastoma. *Cancer Res* **57**: 3772–3778

Maestro R, Dolcetti R, Gasparotto D, Doglioni C, Pelucchi S, Barzan L, Grandi E and Boiocchi M (1992) High frequency of p53 gene alterations associated with protein overexpression in human squamous cell carcinoma of the larynx. *Oncogene* **7**: 1159–1166

Maesawa C, Tamura G, Suzuki Y, Ogasawara S, Sakata K, Kashiwaba M and Satodate R (1995) The sequential accumulation of genetic alterations characteristic of the colorectal adenoma–carcinoma sequence does not occur between gastric adenoma and adenocarcinoma. *J Pathol* **176**: 249–258

Mandai M, Konishi I, Kuroda H, Komatsu T, Yamamoto S, Nanbu K, Matsushita K, Fukumoto M, Yamabe H and Mori T (1998) Heterogeneous distribution of K-ras-mutated epithelia in mucinous ovarian tumors with special reference to histopathology. *Hum Pathol* **28**: 34–40

Reale MA, Hu G, Zafar AI, Getzenberg RH, Levine SM and Fearon ER (1994) Expression and alternative splicing of the deleted in colorectal cancer (DCC) gene in normal and malignant tissues. *Cancer Res* **54**: 4493–4501

Reles A, Schmider A, Press MF, Schonborn I, Huber-Schumacher WFS, Strohmeyer T and Lichtenegger W (1996) Immunostaining of p53 protein in ovarian carcinoma: correlation with histopathological data and clinical outcome. *J Cancer Res Clin Oncol* **122**: 489–494

Reyes-Mugica M, Rieger-Christ K, Ohgaki H, Ekstrand BC, Helie M, Kleinman G, Yahanda A, Fearon ER, Kleihues P and Reale MA (1997) Loss of DCC expression and glioma progression. *Cancer Res* **57**: 382–386

Saegusa M, Kamata Y, Isono M and Okayasu I (1996) Bcl-2 is correlated with a low apoptotic index and associated with progesterone receptor immunoreactivity in endometrial carcinomas. *J Pathol* **180**: 275–282

Shibata D, Reale MA, Lavin P, Silverman M, Fearon ER, Steele G, Jr, Jessup JM, Loda M and Summerhayes IC (1996) The DCC protein and prognosis in colorectal cancer. *N Engl J Med* **335**: 1727–1732

Sinicrope FA, Ruan SB, Cleary KR, Stephens LC and Lee JJ (1995) bcl-2 and p53 oncoprotein expression during colorectal tumorigenesis. *Cancer Res* **55**: 237–241

Slamon DJ, Godolphin W, Jones LA, Holt JA, Wong SG, Keith DE, Levin WJ, Stuart SG, Udove J, Ullrich A and Press MF (1989) Studies of HER-2/*neu* proto-oncogene in human breast and ovarian cancer. *Science* **244**: 707–712

Uchino S, Tsuda H, Noguchi M, Yokota J, Terada M, Saito T, Kobayashi M, Sugimura T and Hirohashi S (1992) Frequent loss of heterozygosity at the DCC locus in gastric cancer. *Cancer Res* **52**: 3099–3102

Wynford-Thomas D (1992) p53 in tumour pathology: can we trust immunohistochemistry? *J Pathol* **166**: 329–330

Zeimet AG, Widschwendter M, Uhl-Steidl M, Muller-Holzner E, Daxenbichler G, Marth C and Dapunt O (1997) High serum levels of soluble CD44 variant isoform v5 are associated with favourable clinical outcome in ovarian cancer. *Br J Cancer* **76**: 1646–1651

*© 2000 Cancer Research Campaign*
